# Thinking before sinning: reasoning processes in hedonic consumption

**DOI:** 10.3389/fpsyg.2014.01268

**Published:** 2014-11-04

**Authors:** Jessie de Witt Huberts, Catharine Evers, Denise de Ridder

**Affiliations:** ^1^London School of Hygiene and Tropical MedicineLondon, UK; ^2^Department of Clinical and Health Psychology, Utrecht UniversityUtrecht, Netherlands

**Keywords:** self-licensing, indulgence, justification, health behavior

## Abstract

Whereas hedonic consumption is often labeled as impulsive, findings from self-licensing research suggest that people sometimes rely on reasons to justify hedonic consumption. Although the concept of self-licensing assumes the involvement of reasoning processes, this has not been demonstrated explicitly. Two studies investigated whether people indeed rely on reasons to allow themselves a guilty pleasure. Participants were exposed to a food temptation after which passive and active reasoning was assessed by asking participants to indicate the justifications that applied to them for indulging in that temptation (Study 1) or having them construe reasons to consume the hedonic product (Study 2). Regression analyses indicated that higher levels of temptation predicted the number of reasons employed and construed to justify consumption. By providing evidence for the involvement of reasoning processes, these findings support the assumption of self-licensing theory that temptations not only exert their influence by making us more impulsive, but can also facilitate gratification by triggering deliberative reasoning processes.

## INTRODUCTION

Many modern day health problems such as obesity, Type 2 diabetes, cardiovascular diseases, hypertension, and certain forms of cancer originate from hedonic overconsumption, that is, the consumption of appetitive but unhealthy products such as nicotine, alcohol, and fatty or sweet snacks. As such, hedonic products tend to invoke conflict in most people: appealing to our indulgent inclinations while simultaneously signaling a breach of our long-term goals (e.g., Ainslie, 1975; Wertenbroch, 1998). Thus, people may inherently be more inclined to pursue the hedonic option, yet will only do so when the situation allows them to justify the violation of personal standards or goals (Kivetz and Simonson, 2002; Khan and Dhar, 2006; Kivetz and Zheng, 2006). Consequently, people are motivated to find reasons that justify abandoning their self-set rules and goals. As such, confrontation with tempting hedonic products may not solely elicit impulsive actions as is conceptualized in many models of self-regulation but may also elicit reasoning processes. Such self-licensing, or the tendency to rely on reasons and arguments to justify indulgence, challenges the dual-process view dominating the self-regulation literature. These so-called dual-process models of self-regulation (e.g., Metcalfe and Mischel, 1999; Strack and Deutsch, 2004) describe temptations as hot, impulsive and automatic forces that need to be counteracted by rational and controlled processes, such as reasoning (De Witt Huberts et al., 2014). Although self-licensing assumes the involvement of reasoning processes, this has not yet been demonstrated explicitly. The aim of the current paper is therefore to establish whether people indeed deliberate to allow themselves an otherwise guilty pleasure, thereby providing evidence for the involvement of reasoning processes in indulgent behavior.

## SELF-LICENSING

The concept of self-licensing is based on findings from decision-making research that people are more likely to make a choice that can easily be justified (Shafir et al., 1993). As the need to choose often creates conflict, decision makers seek and construct reasons in order to resolve the conflict and justify their choice (e.g., Simonson, 1989; Shafir et al., 1993; Kivetz, 1999). When confronted with a typical self-regulation dilemma of gratifying immediate desires versus the pursuit of long term benefits, people will in many cases be inclined to pursue the hedonic option, but will be less likely to do so when the situation makes it difficult for them to justify it (Kivetz, 1999; Okada, 2005). Thus, sometimes indulgence is not determined by ones’ capacity to control oneself, but rather by the availability of reasons that one has to justify the prospective indulgence (e.g., De Witt Huberts et al., 2014).

Self-licensing processes in self-regulation have been afforded some attention in the domain of moral behavior, where people whose past behavior (e.g., acting non-prejudiced) provides them with some kind of moral credentials that license them to subsequently behave in a way that violates these principles (e.g., voicing prejudiced opinions; Monin and Miller, 2001; Effron and Monin, 2010). In recent years the empirical evidence accumulated indicates that self-licensing processes also contribute to indulgent behavior, demonstrating that providing people with a justification, such as effort (Kivetz and Zheng, 2006), achievement (Mick and Faure, 1998; Kivetz and Zheng, 2006), altruism (Khan and Dhar, 2006), or prior restraint (Mukhopadhyay and Johar, 2009) leads to a preference for hedonic over functional choice (e.g., Khan and Dhar, 2006; Kivetz and Zheng, 2006) as well as hedonic overconsumption (De Witt Huberts et al., 2012; Taylor et al., 2014). For example, participants who were under the impression of having exerted more effort consumed more hedonic snacks compared to participants believing their equally exerted effort did not exceed the norm (De Witt Huberts et al., 2012). On a more indirect level, a study on compensatory beliefs found that compensatory intentions (“I eat this cookie now, but I cut back later”) – which could be seen as a sort of justification – are associated with the decision to indulge amongst dieters (Kronick and Knäuper, 2010).

While these studies provide evidence for the importance of reasons in indulgent behavior, the assumed justification processes in these studies have not been demonstrated explicitly. In studies investigating self-licensing processes, the backbone of the process – seeking and constructing reasons to justify prospective indulgent behavior – has remained implicit. For instance, in the aforementioned studies participants were provided with, rather than having to construct a reason that justified subsequent hedonic consumption. What’s more, the provided justifications remained implicit, for example by making participants think they did a certain task twice rather than explicitly alluding to the extra effort they exerted (De Witt Huberts et al., 2012), or by presenting the licensing cue and consumption as being unrelated in two separate tasks (e.g., Kivetz and Zheng, 2006; Mukhopadhyay and Johar, 2009). It has even been illustrated that self-licensing processes can occur by relying on some kind of heuristic (i.e., “I deserve a treat after effort”) or without awareness (e.g., Khan and Dhar, 2006; Kivetz and Zheng, 2006).

Whilst these studies convincingly demonstrate how engrained self-licensing is in our behavioral repertoire, seemingly relying on heuristics and learned automatic associations, to our knowledge it has not yet been demonstrated whether being confronted with a temptation can indeed induce seeking and construction of justifications, thereby tempering the assumption that self-licensing is a reasoned process. To date, only two studies have attempted to explicitly capture the reasoning processes involved in justifying indulgence (Khan and Dhar, 2006; Mukhopadhyay and Johar, 2009), yielding mixed results: one study finding that the justifications people put forward mediated the relationship between prior restraint and indulgent choice (Mukhopadhyay and Johar, 2009; Study 1) while another study found that people were not aware of applying justifications to indulge (Khan and Dhar, 2006; Study 2). More importantly, besides these contradicting results, both studies inquired into the use of justifications after the indulgence had taken place, thereby hindering conclusions about *a priori* deliberation processes that would facilitate gratification, and leaving open the possibility that participants were applying a justification in hindsight as dissonance reduction (Festinger, 1957) or reporting a general belief that indulgent behaviors need to be justified (Xu and Schwarz, 2009).

This lack of explicit evidence for the justification process is surprising, not only as introspection tells us that we sometimes actively seek and construct reasons when confronted with a tempting choice (e.g., Mick and DeMoss, 1990). But also, and more importantly, the very process of self-licensing, applying a reason that justifies a departure from one’s long term goals, suggests that there must be some active argumentation involved that fosters such a strategic decision. The aim of the current paper is therefore to ascertain justification processes explicitly, thereby providing credence to the observation that deliberative and reflective processes can facilitate indulgent behavior.

To investigate this, we provided weight-conscious participants with an attractive but goal-threatening product, a chocolate bar, and measured passive as well as active engagement in reasoning behavior. We hypothesized that when the lure of the temptation, and thus the need for justification, is larger, the more likely people are to engage in justification processes. More specifically, we predict that the more one is tempted by the ‘forbidden’ product, the more reasons one will employ to justify subsequent consumption (Study 1). Moreover, we predict that exposure to a temptation will not only stimulate employment of reasons available, but will lead to active construction of justifications (Study 2).

## STUDY 1

### METHOD

#### Participants

Sixty female university students participated in this study for course credit or 4€. Female participants were recruited as they experience food more as a self-regulatory dilemma than males (Grogan et al., 1997). This makes them more likely to use justifications for indulging in highly caloric food. This assumption was corroborated by the finding that all participants responded positively to the question: “Are you currently watching you weight?” One participant who was an outlier (SD > 3) on the independent variable (i.e., Temptingness of the hedonic product) and one who did not comply with the instructions were removed resulting in a final sample of 58 participants with a mean age of 20.21 (SD = 2.02).

***Ethics statement***. The study was conducted in accordance with the ethical standards described by the Medical Research Involving Human Subjects Act (WMO, 2012). This Act exempts research on healthy human subjects from review for as long as it does not involve any invasion of participants’ integrity. Consequently, no formal ethical approval was required according to Dutch national standards. Written consent was required from each participant prior to participation.

### PROCEDURE

The study was presented as a marketing study for a large retail concern conducted by the university’s business school. The participants were seated behind a table with the hedonic snack product (a luxurious chocolate bar) that remained covered until participants were instructed to remove the cover to evaluate the product. The goal and purpose of the study were presented in a booklet, explaining that the producer, as part of the market introduction of a new product, was interested in the evaluation of this product by the target group of students aged 18–30. The participants had to indicate how tempting they found the snack, among other filler items assessing the attractiveness and their willingness to try the product. After completing this part of the evaluation, the participants then read instructions that the snack was intended as an indulgence and that, as information for the marketing strategy, the producers wanted to know when and for what reasons the target group would allow themselves this particular hedonic snack. On a subsequent page the participants could indicate the reasons for having this product that applied to them out of a list of random reasons. It was explicitly alluded that they could tick of as little or many reasons as long as they applied to them. Finally, demographic variables were asked before participants were debriefed and reimbursed for participation. A suspicion probe asking participants to write down what they thought the purpose of the study was, revealed that the cover story had been successful and that none of the participants had been aware of the real question under study.

### MATERIALS

#### Hedonic product

In line with the cover story the temptation consisted of a recently launched luxurious chocolate bar by a well known brand.

#### Temptingness

Participants were asked to indicate to what extent they perceived the product as tempting on a seven point Likert scale ranging from 1 (*not at all*) to 7 (*very much*). This item was presented among filler items assessing how likely they were to buy the product, how much they were willing to pay for the product, and how willing they were to try the product.

#### Hunger

As food consumption is largely determined by hunger and appetite, participants had to indicate their levels of hunger and appetite on seven point Likert scales (1 *not at all* to 7 *a lot*) that were combined into a single measure of Hunger, (Cronbach’s α = 0.88) to control for the effect of hunger on the temptingness of the product.

#### Justifications

As an explicit measure of the justification processes involved in self-licensing, participants could indicate the reasons that applied to them for indulging in the hedonic product out of a list 30 reasons. The list of justifications consisted of variations of well-known justification cues such as effort (Kivetz and Zheng, 2006; De Witt Huberts et al., 2012), achievement (Mick and DeMoss, 1990; Kivetz and Zheng, 2006), and altruism (Khan and Dhar, 2006). Examples of justifications are: “Because I have had a busy period behind me”; “Because I have something to celebrate” and “Because I feel bad today.” Visceral factors that may be used as a reason to consume the product, such as appetite and hunger were not included, as these factors physiologically determine food intake and constitute a biological necessity to consume the hedonic product, rather than a justification. Participants were also provided with the opportunity to add a reason if they had a reason to indulge that was not included in the list. The sum of justifications was used as a measure of reasoning to indulge.

### RESULTS

#### Descriptives

**Table 1** shows the means, SDs and intercorrelations of the variables under study. Generally the participants rated the chocolate bar as tempting (*M* = 5.88, SD = 0.73) with scores ranging from 3 to 7. Participants indicated on average 12.22 (SD = 6.64) reasons to consume the hedonic chocolate bar. None of the participants added a reason that was not yet included in the list. The most frequently utilized reasons were: “I have something to celebrate” (65%); “I have exerted effort for something important” (58%), and “I deserve a reward”(56%).

**Table 1:** Means, SDs, and Correlations (Study 1).

	1	2	3
Hunger (1)	–		
Temptingness (2)	0.19	–	
Number of justifications (3)	0.08	0.31^∗^	–
*M*	3.74	5.88	12.22
SD	1.49	0.73	6.64

*Significant at p < 0.05 level.

#### Main analysis

A hierarchical regression analysis was performed to determine whether temptingness predicted the number of justifications participants employed to justify consumption. In the first step hunger was included as a control variable. In the second step temptingness was entered as predictor. As can be seen in **Table 2**, the first step did not reach significance, *p* = 0.54. In the second step temptingness of the hedonic product significantly predicted the number of reasons, *p* = 0.02, explaining 9.7% of the variance (unadjusted). The hypothesis that temptingness predicted the application of justifications was therefore confirmed.

**Table 2:** Hierarchical multiple regression analysis for number of justifications (Study 1).

	Number of justifications		
	β	ΔF	ΔR^2^
*Step 1*		0.39	0.01
Hunger	0.08		
*Step 2*		5.51^∗^	0.09
Temptingness	0.31^∗^		

*Significant at p< 0.05 level.

### DISCUSSION

The results of Study 1 confirmed that subjective evaluation of temptation strength predicts the employment of justifications. These results suggest that reasoning processes that could facilitate gratification may already take place before prospective indulgence. Whilst the current studies explicitly demonstrate the justification processes people employ when confronted with temptation, a limitation is that participants were provided with justification cues, not allowing any conclusions about self-generated justifications and thus actual active reasoning behavior in the face of temptation. This limitation was addressed in Study 2, thereby allowing to more stringently test whether exposure to temptation indeed elicits active reasoning processes.

## STUDY 2

To investigate whether temptations elicit active engagement in reasoning processes, Study 2 required participants to construe reasons to justify prospective indulgence. In addition, to provide a more stringent test of our assumption that the hedonic product represented a motivational conflict for our participants, we only included female participants who indicated that the chocolate temptation constituted a threat to their personally relevant long-term goal (i.e., weight management).

Conform our hypothesis that temptations can encourage reasoning processes, we predict that higher levels of temptation would be associated with a higher number of reasons construed to consume the tempting threat.

### METHOD

#### Participants

Thirty-seven female university students participated in this study for course credit or 4leela. Again only female participants were recruited as they are more likely to perceive a chocolate bare as a guilty pleasure (Grogan et al., 1997). This assumption was corroborated during the study by including questions asking how much importance the participants assigned to watching their weight and how much the chocolate bar constituted a threat to their weight. Only participants who assigned high importance to their weight and found the product to be interfering with this goal (scoring >3 on both scales ranging from 1 to 7) were included in the final analyses. One participant who did not meet the criteria was excluded from analyses, resulting in a sample of 36 students with a mean age of 19.63 (SD = 4.07).

***Ethics statement***. See Study 1. All participants provided written informed consent.

#### Procedure

The procedure was similar to the one employed in Study 1 except that this time participants were asked to actively come up with personally relevant reasons that would allow them to consume the hedonic product. Afterward, together with filler items such as frequency of buying and consuming snacks, participants were asked to indicate how relevant weight management was to them and how threatening they perceived the chocolate bar to be to this goal, to ensure that licensing was indeed necessary. Finally, demographic variables were asked before participants were debriefed and reimbursed for participation. A suspicion probe asking participants to write down what they thought the purpose of the study was, revealed that the cover story had been successful and that none of the participants had been aware of the real question under study.

### MATERIALS

#### Hedonic product

The tempting product was similar to the one used in Study 1.

***Hunger***. Again the mean scores of hunger and appetite were combined into a Hunger score (Cronbach’s α = 0.88) to serve as control variable.

#### Temptingness

Participants had to indicate how tempting they perceived the product to be on a seven point Likert scale ranging from 1 (*not at all*) to 7 (*very much*). This item was presented among filler items assessing how likely they were to buy the product and how much they were willing to pay for the product.

#### Justifications

Instead of being provided with justifications, participants were asked to write down the reasons they would have to consume the tempting product. Similar to Study 1, participants read the instruction that the retail concern was interested in the reasons people have to eat an indulgent product and were given two examples of such reasons. The participants could then write down as many or as little reasons as they could come up with to subsequently consume this product. Again it was emphasized that it did not matter how many reasons they came up with, as long as they were personally relevant. For similar reasons as in Study 1, visceral reasons such as hunger or appetite were not included in the final score. The total number of reasons participants came up with to consume the product was used as an indicator of reasoning to indulge.

#### Goal relevance

To control for goal relevance, participants were asked to indicate how important weight management was for them (“Do you watch your weight?”) and how threatening they deemed the chocolate product to be to their weight management goal (“How bad is the product for maintaining your ideal weight”) on seven point Likert scales ranging from 1 (*not at all*) to 7 (*very much*).

### RESULTS

#### Descriptives

**Table 3** shows the means, SDs and intercorrelations of the variables under study. 16.77% of the total amount of self-generated reasons involved hunger or appetite (e.g., “because I have a chocolate craving”; “because I’m hungry”) and were not included in the measure of justification. The following analyses are thus based on the remaining 83.23% of the self-generated reasons that actually constituted justifications. Participants on average came up with 3.17 (SD = 2.55) justifications to consume the hedonic chocolate bar. Examples of reasons are “After a day of studying hard”; “Because I have finished/passed my midterms; “To make it a special evening with my (boy)friend.” The mean score of temptingness was 5.55 (SD = 1.65), showing a wide variety of scores ranging from 1 (*very low*) to 7 (*very high*). The participants attached a medium to high importance to achieving and maintaining a healthy weight, 4.9 (SD = 1.14) and considered the chocolate product as interfering with that goal with a mean score of 6.5 (SD = 0.65) on how bad for weight management they perceived the chocolate product to be.

**Table 3:** Study 2: means, SDs, and Correlations (Study 2).

	1	2	3	4	5
Hunger (1)	–				
Temptingness (2)	0.01	–			
Number of justifications (3)	0.06	0.36^∗^	–		
Weight watching importance (4)	-0.27	0.29	0.31	–	
Product bad for weight (5)	-0.23	0.21	0.21	0.38^∗^	–
*M*	4.01	5.56	3.17	4.89	6.5
SD	1.60	1.65	2.55	1.14	0.65

*p < 0.05.

#### Main analysis

A hierarchical regression analysis was performed to determine whether temptingness predicted the number of reasons participants constructed to justify consumption. In the first step hunger was included as control variable. In the second step temptingness was entered as predictor. As can be seen in **Table 4**, the first step did not reach significance, *p* = 0.75. In the second step, subjective temptingness of the hedonic product significantly predicted the number of reasons participant construed, *p* = 0.03, explaining 13.2% of the variance (unadjusted). Participants who were more tempted by the chocolate product constructed more reasons to indulge.

**Table 4:** Hierarchical multiple regression analysis for number of self-generated justifications (Study 2).

	Number of justifications		
	β	ΔF	ΔR^2^
*Step 1*		0.11	0.00
Hunger	0.06		
*Step 2*		4.88^∗^	0.13
Temptingness	0.36^∗^		


*Significant at *p*< 0.05 level.

### DISCUSSION

Study 2 demonstrated that when tempted, people actively construe reasons and justifications to indulge in that guilty pleasure. To our knowledge this study constitutes the first demonstration of actively engaging in justification processes when confronted with a self-regulation dilemma, lending further support to the concept of self-licensing.

## GENERAL DISCUSSION

The current studies established explicit self-licensing processes and demonstrate that people not only apply justifications made available to them to indulge, but they also actively construe justifications in the face of temptation, thereby suggesting that temptations not only exert their power by eliciting impulsive reactions, but also induce reasoning processes that may facilitate indulgent behavior. These results challenge the prevalent idea that deliberation and reflection always foster goal-directed behavior by allowing us to overcome the stimulus control of temptations, as is suggested by many dual-process models of self-regulation (e.g., Metcalfe and Mischel, 1999; Strack and Deutsch, 2004). Instead, these findings indicate that temptations also can exert their influence via the reflective or ‘cool’ system, suggesting that hedonic behavior is not exclusively the result of impulsive processes. The current results thus offer a novel point of view for the conceptualization of hedonic behavior, that may have a familiar appeal to many of us, yet is not incorporated in the main theories of hedonic (over)consumption (e.g., Baumeister and Heatherton, 1996; Metcalfe and Mischel, 1999; Strack and Deutsch, 2004). Future research should incorporate previous findings on self-licensing with the current evidence for explicit justification processes, to establish to what extent the observed reasoning processes can stimulate actual indulgent behavior.

Despite this novel contribution, a few issues remain to be explored. Firstly, no actual hedonic behavior was assessed, so it is not clear to what extent the observed licensing processes translate into actual indulgent consumption. As human behavior is influenced by many factors simultaneously, it could very well be that additional factors inhibit the premeditated indulgent behavior ultimately. Nevertheless, prior research into self-licensing has already demonstrated that even being provided with a single reason can facilitate indulgent behavior, it thus seems likely that the construction of justifications as is currently observed would produce similar effects. Relatedly, the current studies used the number of reasons as a quantification of justification processes, it remains to be seen, however, if the number of reasons is the crucial connection between justification and behavior. To discern whether the quantity, the quality, or an interaction between the two, is decisive for the translation from justification to indulgence is something that should be explored in future studies. Even so, the current studies provide a valuable contribution to the concept that temptations not only elicit impulsive automatic reactions, but also stimulate reasoning processes.

A limitation of the current studies is that the justifications participants had to give were hypothetical, that is, they had to indicate what would be a justification for them to consume that product, but they did not have to justify actual consumption at that moment itself. However, asking participants to justify consumption *in situ*, might have elicited reactivity or eluded social desirable answers, as justifications can have the negative connotation of being excuses for one’s undesirable behavior, something people presumably do not like to exhibit. Inquiring specifically after the justifications people tend to use as part of a consumer study, thereby acknowledging that it is a common process, probably allowed for a more free and honest reflection of the justifications people apply. Similarly, the current studies did not establish spontaneous justification processes, but required the participants to come up with justifications. We deem it quite difficult, however, to establish explicit self-licensing processes spontaneously, as again this would be burdened by the inhibiting influence of social desirability when having to explicitly name the justifications one uses. It seems more likely that the self-licensing processes people rely on are intrapersonal in their nature and take the form of self-talk or licensing thoughts, rather than explicitly stating the justification. The latter could emphasize the sometimes inconsistent justifications people rely on, thereby challenging their power. Nevertheless, even seeing a strong effect of temptation strength on the justifications people apply and construe when the prospective consumption is hypothetical and under the social constraints posed by a lab study, suggests that the effect may even be stronger in daily hedonic behavior, where one mainly has to justify one’s goal-violating behavior to oneself and not necessarily to others.

A second limitation is the cross-sectional nature of the current findings. Temptation strength should be manipulated in future studies to establish their causal role in the justification process. This however, raises the question of how temptingness could be manipulated adequately without eradicating the temptation strength altogether. Possibilities are manipulating temptation strength itself (e.g., weak and strong temptations; Kroese et al., 2011) or varying the degree of goal threat a temptation constitutes. Despite the lack of causal inferences, the current studies have the advantage that subjective temptation was assessed, thus capturing the seductive power of the temptations more accurately, as temptations tend to be idiosyncratically determined rather than generally established; something that is also reflected by the varying ratings of temptingness in the present studies of a universally acknowledged temptation such as chocolate.

A third limitation of the study is that both studies included a relatively low number of participants with low statistical power as a result. Before any firm conclusions can be drawn, replication of the findings in larger samples is warranted. Replication should also include participants from non-student community samples to determine the extent to which justification processes are associated with the typical characteristics of young and well educated people.

Despite the issues in need of further exploration, the finding that self-licensing in fact involves reasoning processes brings new light to the conventional conceptualization that indulgent behavior is the result of impulsive processes, while reasoning processes would support goal directed behavior. Not only do the current results, together with prior findings on self-licensing, suggest that reasoning processes can instigate hedonic behavior, they also indicate that confrontation with temptation not always prompts automatic reflexive behaviors. Although it has been demonstrated that temptations not necessarily lead to gratification, but that confrontation with temptation can also automatically reinstate ones’ long term goal (e.g., Fishbach et al., 2003; Kroese et al., 2009), these findings demonstrate that temptations do not always elicit automatic reactions, but can also induce reflective processes that could contribute to indulgent behavior. Together these results suggest that the line between ‘hot’ and ‘cool’ processes is not as clear-cut. By uncovering alternative pathways to hedonic overconsumption, we hope to contribute to a more comprehensive view of health behavior that goes beyond the traditional divide between the passions and reason.

## Conflict of Interest Statement

The authors declare that the research was conducted in the absence of any commercial or financial relationships that could be construed as a potential conflict of interest.
